# The impact of dietary interventions on cardiometabolic health

**DOI:** 10.1186/s12933-025-02766-w

**Published:** 2025-05-31

**Authors:** Erind Gjermeni, Raluca Fiebiger, Linnaeus Bundalian, Antje Garten, Torsten Schöneberg, Diana Le Duc, Matthias Blüher

**Affiliations:** 1 Department of Cardiology, Median Center for Rehabilitation Schmannewitz, 04774 Dahlen, Germany; 2 Institute of Human Genetics, University Medical Center Leipzig, 04103 Leipzig, Germany; 3 Pediatric Research Center, University Hospital for Children and Adolescents, Leipzig University, 04103 Leipzig, Germany; 4 Rudolf Schönheimer Institute of Biochemistry, Molecular Biochemistry, Medical Faculty, University of Leipzig, Leipzig, Germany; 5 School of Medicine, University of Global Health Equity, Kigali, Rwanda; 6 Department of Genetics, Center for Diagnostics at Chemnitz Clinics, 09116 Chemnitz, Germany; 7 Department of Evolutionary Genetics, Max Planck Institute for Evolutionary Anthropology, 04103 Leipzig, Germany; 8 Helmholtz Institute for Metabolic, Obesity and Vascular Research (HI-MAG) of the Helmholtz Zentrum München at the University of Leipzig and University Hospital Leipzig, 04103 Leipzig, Germany

## Abstract

Obesity and cardiometabolic diseases are leading causes of morbidity and mortality among adults worldwide. These conditions significantly contribute to and exacerbate other major causes of illness and death, including cancer, neurodegenerative diseases, and chronic kidney disease. The growing burden of these diseases has increased the interest of modern medicine in understanding metabolic processes and health, with diet emerging as a pivotal modifiable factor, alongside physical inactivity and smoking. In this review, we discuss the pathophysiological and evolutionary foundations of metabolic processes that may link “unhealthy” nutrition to obesity and cardiometabolic diseases and review the current literature to assess the effects of various diet interventions and patterns on cardiometabolic parameters. Special emphasis is placed on summarizing the latest, albeit partially contradictory, evidence to offer balanced dietary recommendations with the ultimate aim to improve cardiometabolic health.

## Background

Cardiometabolic diseases (CMD) are closely linked to dietary habits and obesity, currently ranking as the leading causes of morbidity and mortality among adults worldwide [1]. The pandemic proportions of this disease cluster result not only in adverse health outcomes but also in staggering economic costs that place immense strain on healthcare budgets [2, 3]. Cardiovascular diseases (CVD), the primary contributors to global mortality, are at least theoretically preventable through effective management of behavioral and environmental risk factors. Among these, an unhealthy diet is the most significant behavioral risk factor, followed by physical inactivity and smoking [4, 5]. Furthermore, CMD substantially contribute to other major causes of global morbidity and mortality [6, 7], including cancer [7], dementia and neurodegenerative diseases [8], type 2 diabetes mellitus (T2DM) [9], and chronic kidney disease [10].

**Fig. 1:**
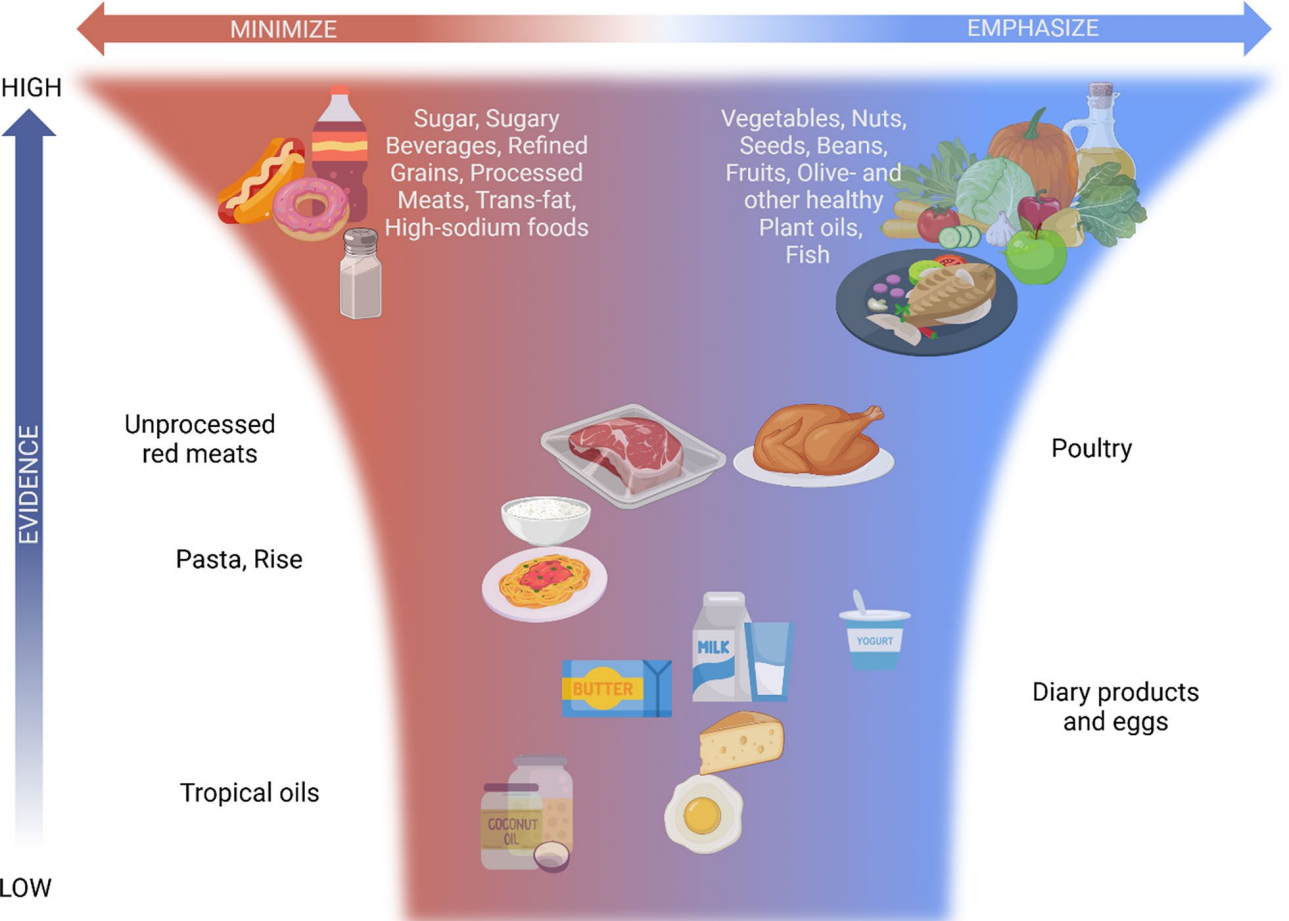
Central illustration. General dietary recommendations for cardiometabolic health. Visual representation of common foods and the level of general consensus regarding the strength of evidence (foods in the upper part have wide consensus), as well as the general recommendations (foods on the right should be emphasized in the diet)

Metabolism—the process that makes the energy from food accessible for sustaining life—has gained increasing attention in modern medicine due to its central role in chronic diseases, as well as its relevance to sports medicine and performance [11]. This growing interest stems from the recognition that metabolic processes not only regulate energy balance, but also influence inflammation, immune function, cellular health, and physical performance [11]. Understanding how dietary components impact metabolic pathways could unlock new prevention and treatment strategies for CMD (Fig. 1).

The evaluation of metabolic flexibility, insulin sensitivity, and metabolic regulation has also become an area of increasing interest [12, 13]. Recently, a novel multimarker indicative of metabolic vulnerability (i.e. six serum biomarkers including GlycA, small HDL, valine, leucine, isoleucine, and citrate concentrations) demonstrated remarkable accuracy in predicting all-cause mortality, particularly in patients with CVD [14–16]. The assessment of metabolic health and performance, through direct and indirect measures of mitochondrial function and metabolic flexibility, is widely utilized for evaluating sports performance, fitness levels, and CMD status. However, these methods are often too invasive and complex for routine use in clinical settings [11, 17]. Fortunately, more accessible biomarkers, discussed in the following paragraph, offer reliable assessments of cardiometabolic health (CMH) [17–19].

### Biomarkers of cardiometabolic health

**Fig. 2:**
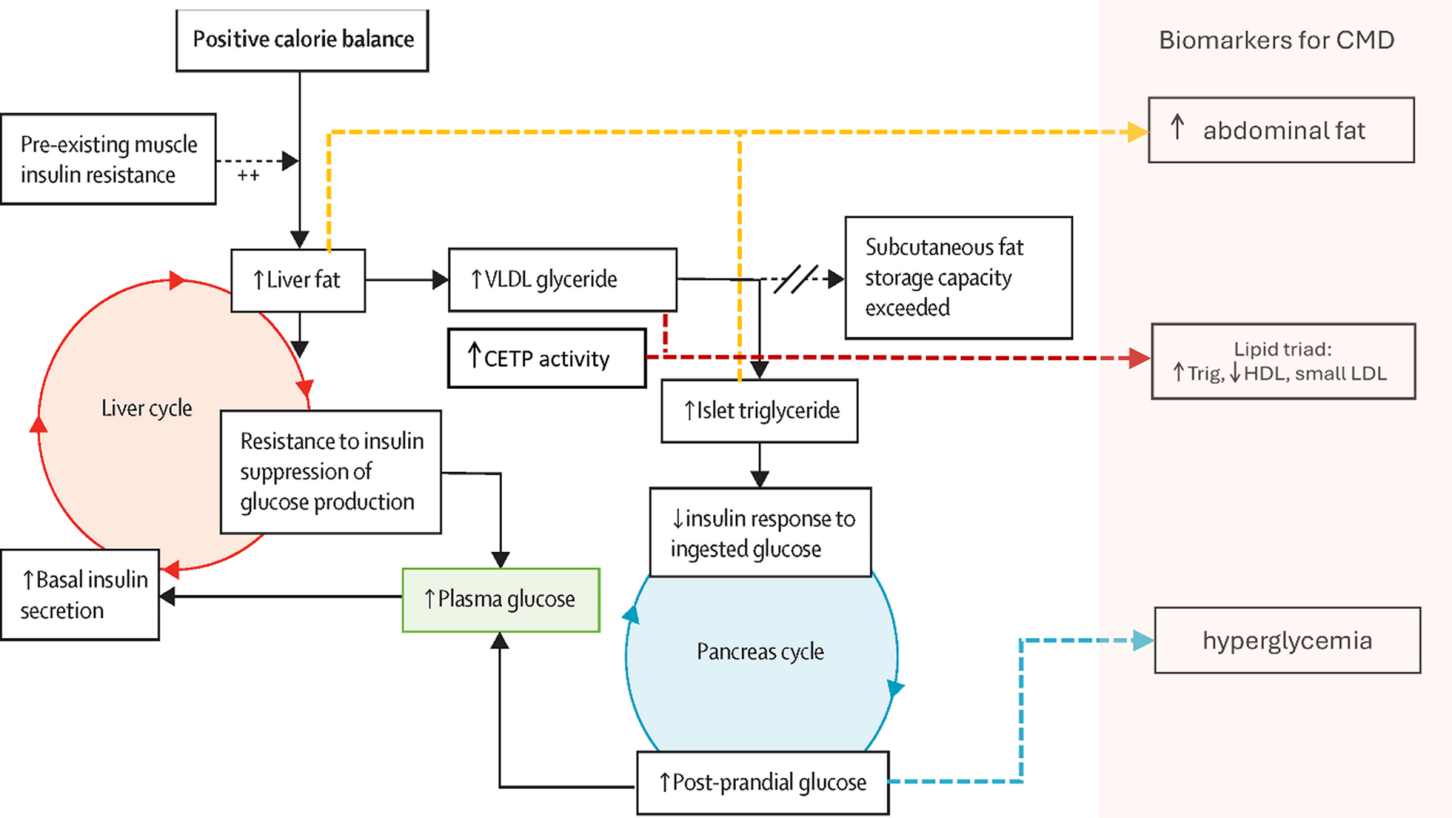
Pathophysiological background of the clinical biomarkers (adapted from ref [20]). During excess calorie intake, de novo lipogenesis processes carbohydrates that cannot be stored as glycogen, promoting fat accumulation in the liver. Because this process is stimulated by insulin, individuals with some degree of insulin resistance (determined by genetic and lifestyle factors) accumulate liver fat more quickly due to higher insulin concentrations. Increased liver fat further exacerbates resistance to the suppression of hepatic glucose production by insulin. A small increase in plasma glucose triggers increased insulin secretion to maintain euglycemia. This resulting hyperinsulinemia further enhances the conversion of excess calories into liver fat, creating a vicious cycle. Fatty liver leads to an increased export of VLDLs into the circulation, which promotes fat delivery to all tissues, including the pancreas. Moreover, high levels of VLDL, in concert with increased CETP activity, result in the enrichment of LDL and HDL with triacylglycerols, while depleting their cholesterol content. This leads to the typical lipid triad: high triacylglycerols, low HDL cholesterol, and small LDL particles. Excess fatty acids in the pancreatic islets can impair insulin secretion in response to ingested food, leading to postprandial hyperglycemia. The hyperglycemia further increases insulin secretion, which consequently elevates hepatic lipogenesis, speeding up the liver cycle and driving the pancreas cycle. Eventually, the inhibitory effects of fatty acids and glucose on the islets reach a threshold, triggering β-cell failure and a fairly sudden onset of clinical diabetes. Adapted from ref. [21], with permission from Elsevier

CMH refers to the health of the cardiovascular and metabolic systems. It is influenced by multiple risk factors that collectively determine the likelihood of developing CVD, T2DM, and other related conditions. A practical evaluation of CMH can be achieved by assessing adiposity, blood glucose levels, blood lipid profiles, blood pressure, and the presence or absence of clinical CVD [18, 19]. Skeletal muscle plays a central role in energy metabolism, as approximately 80% of postprandial carbohydrate oxidation occurs in skeletal muscle [11, 20–22]. Insulin resistance in skeletal muscle can emerge decades before the onset of β-cell failure [11, 20–22]. Chronic positive energy balance tends to elevate glucose levels, prompting increased insulin production. Hyperinsulinemia suppresses hepatic glucose production and shifts liver metabolism toward the conversion of carbohydrates into fat [23]. This results in increased hepatic lipid production (e.g., triacylglycerols, commonly called triglycerides), fat accumulation in the liver, and ultimately the development of fatty liver and hepatic insulin resistance (Fig. 2).

The worsening of insulin resistance further escalates insulin production and enhances carbohydrate-to-lipid conversion in the liver, initiating a vicious cycle. Excess fat in the liver can lead to increased lipid export throughout the body, including deposition in abdominal organs—a hallmark of metabolic dysfunction—once subcutaneous fat storage capacity is exceeded. Pancreatic fat accumulation may impair insulin production, eventually leading to T2DM in susceptible individuals.

In this state, elevated cholesteryl ester transfer protein (CETP) activity enriches low-density lipoprotein (LDL) and high-density lipoprotein (HDL) particles with triacylglycerol [24] while depleting their cholesterol content [25]. Hepatic lipase, upregulated by hyperglycemia [24, 26] and exhibiting increased activity in insulin-resistant states [27], rapidly metabolizes triacylglycerol-rich HDL and LDL. This process forms small HDL particles, which are cleared more quickly, and small LDL particles, which are highly atherogenic. This contributes to the characteristic lipid triad of insulin resistance: (1) elevated plasma triacylglycerols, (2) reduced HDL cholesterol levels, and (3) the presence of highly atherogenic small LDL particles. This triad is a strong predictor of poor metabolic health.

On the other hand, improving metabolic health through a healthy lifestyle has proven highly effective in reducing CVD mortality. A striking example is the community-wide intervention in North Karelia, Finland. In the late 1960s, men in North Karelia had the highest CVD mortality rate in the world. Through comprehensive national policies and health promotion initiatives across Finland, the population achieved significant reductions in cholesterol levels, blood pressure, and smoking rates. Over 35 years, age-adjusted coronary heart disease (CHD) mortality rates decreased by 85% in North Karelia and by 80% nationwide among men aged 35–64 years [28–30]. These findings underscore that dietary and lifestyle interventions can yield substantial improvements in CMH.

### Evolutionary insights and cardiometabolic health

Medicine in the light of evolution provides a valuable framework for understanding human health and disease by applying principles of evolutionary biology. The mismatch theory suggests that many modern diseases arise because human bodies are adapted to past environments, not the rapidly changing conditions of contemporary life. Our ancestors evolved to thrive on diets, activity levels, and stressors vastly different from today’s fast-paced, sedentary lifestyle dominated by processed foods. This mismatch helps explain the rise in conditions like obesity, T2DM, and CVD, as our evolutionary adaptations are poorly suited to modern environments [31, 32]. For instance, the “thrifty genotype” hypothesis posits that traits evolved to support survival during periods of food scarcity now predispose individuals to obesity and related metabolic conditions in today’s environment of caloric abundance [32]. Inflammation in CVD may also have evolutionary roots. High infectious burdens in ancestral populations likely selected for strong inflammatory responses, but in modern contexts, these pro-inflammatory genes may contribute to chronic diseases like obesity and CVD. Diet, particularly the consumption of highly processed foods rich in refined sugars, is a major driver of inflammation today. Insights into ancestral diets, enabled by next-generation sequencing, proteomics, and isotope analysis, are critical to understanding these evolutionary dynamics.

Diet has long been proposed as a key factor in hominin evolution, particularly in brain size expansion. Findings from a 3.5-million-year-old *Australopithecus* site suggest that these hominins had herbivore-like diets, distinct from carnivores, indicating that meat consumption was not a driving factor in the evolution of larger brains [33]. Dental calculus, a mineralized form of dental plaque, offers a direct window into ancient diets [34]. For instance, studies have shown that Neanderthals likely consumed roots, bulbs, and other plant materials, particularly during cooler periods when animal resources may have been less accessible [35, 36]. The analysis of dental calculus from Neanderthal remains has identified starch grains and phytoliths, which suggest a significant plant component in their diet, alongside evidence of meat consumption [35, 37]. However, alternative theories have challenged this view. Bone collagen isotope studies have significantly advanced understanding of Neandertal diets, consistently showing exceptionally high nitrogen isotope ratios. The findings reinforce Neandertals' role as successful top-level carnivores, even after modern humans arrived in Europe. High [15] N values in bone collagen are explained solely by mammal meat consumption, supported by archeological evidence, without requiring alternative explanations such as food processing (fermented food, cooking), specific prey types (mammoths or young mammals), or additional protein sources like fish or mushrooms [38].

Fermented foods contain bacteria-derived D-amino acids and their metabolites, which can regulate human immune functions and energy homeostasis through hydroxycarboxylic acid receptors (HCARs). While most mammals have two HCARs (HCAR1 and HCAR2), humans and other hominids possess a third receptor, HCAR3. Evolutionary and functional evidence suggests that HCAR3 evolved in hominids as a signaling system for bacteria-derived D-amino acids and their metabolites. As HCAR3 is expressed in monocytes and adipocytes it has been speculated that aromatic D-amino acids and their derivatives from fermented fruits act as signals to attract immune cells to the gut and suppress fat breakdown in adipose tissue [39].

In modern humans, dental calculus analysis has similarly revealed a complex dietary profile. For example, Warinner et al. demonstrated that ancient human dental calculus contains evidence of milk consumption, indicating that dairy products were part of the diet at least in certain populations [34]. Furthermore, fossils from Fuyan Cave, South China, dated to about 80,000 years ago, suggest that early modern humans also engaged in diverse foraging strategies that included both cultivated and wild plant species [40]. The comparative analysis of ancient and modern dental calculus can reflect dietary changes over time. For instance, metaproteomic data from 100 archaeological dental calculus samples ranging from the Iron Age to the post-medieval period (eighth century BC to nineteenth century AD) in England detect proteomic evidence of cereals and plant products [41]. Moreover, studies have shown that the microbial profiles of dental calculus differ markedly from those of dental plaque, suggesting that dietary habits have evolved alongside changes in oral microbiomes [42, 43].

While we can gain insight into the composition of diets by examining fossils and dental calculus, the lack of information on their cardiovascular fitness prevents us from correlating nutrition with cardiovascular outcome. To this end, communities of hunter–gatherers, that are currently living in environments closer to the ancestral one, are an important mirror on how nutrition and lifestyle in general impact cardiovascular health.

Modern hunter–gatherer communities, such as the Hadza of Tanzania, provide valuable insights into ancestral nutrition and lifestyle. The Hadza’s diet, rich in unprocessed, foraged foods like fruits, vegetables, tubers, and wild game, combined with high physical activity, results in exceptional cardiovascular fitness. They exhibit low rates of obesity, hypertension, and other metabolic conditions, even in older age [44, 45]. The Mediterranean diet, which shares similarities with the traditional diet of the Hadza has been extensively studied for its cardiometabolic benefits [46–50].

The evolutionary perspective on diet emphasizes the mismatch between modern lifestyles and ancestral adaptations, highlighting the need for dietary patterns that align with our biological heritage. By drawing lessons from early hominins, Neandertals, and contemporary hunter–gatherer populations, we can better understand the role of diet in preventing CMD.

### The role of macronutrient quality and quantity

Carbohydrates, fats, and proteins in the diet serve as chemical building blocks and energy sources for various bodily functions. If not immediately utilized, they are stored for future use. Maintaining a stable body weight requires balancing energy intake with energy expenditure. When energy intake consistently exceeds expenditure, the excess energy is predominantly stored as fat, leading to an increase in body weight [51]. While the quantity of macronutrients in the diet offers valuable information, equally important is food quality and the complex interplay of foods and dietary patterns, on long-term weight control and metabolic health (Central illustration).

### Dietary carbohydrates

Foods containing carbohydrates consist principally of sugars, starches, and dietary fiber. Carbohydrates provide the main energy source for people worldwide. Upon consumption, many carbohydrates are broken down into glucose, galactose and fructose, which are either acutely utilized or transformed into glucose, stored as glycogen in the liver and muscles for later use. Other monosaccharides, such as mannose or ribose, do play a role in metabolic processes (e.g., nucleotide synthesis), but they are not directly used as primary energy sources. The acceptable macronutrient distribution range for carbohydrates is 45–65% of total daily caloric intake [52], but healthy dietary patterns can be outside this range [49]. The role of mono- and disaccharides (simple sugars) and refined grains as a determinant of adverse health outcomes has been clarified, and clear guidelines relating to their restriction issued [53–56].

Across different foods and beverages, those high in simple sugars and refined grains most strongly associate with long-term weight gain [57, 58] and T2DM [59]. The evidence is especially strong for sugary bewerages [58–60]. Simple sugars and refined grains are commonly found in white bread, white rice, white potatoes, refined pastas, chips, sugary beverages, candy, and many bakery products [58, 61]. Conversely, interventional trials show weight loss and improved glycaemia on low-carbohydrate diets [62, 63]; these evidence provides strong arguments that simple sugars should be avoided to optimize weight and metabolic health.

Dietary fibers are mainly compost of non-starch polysaccharides (e.g., cellulose, hemicellulose, pectins) and non-carbohydrate components (e.g., lignin, suberin, cutin). While fibers are not digested or absorbed by the small intestine, they have crucial roles in improving gut function and supporting the microbiome and are associated with multiple health benefits [64]. Credit for the dietary fiber hypothesis is given largely to Denis Burkitt, who had more than 50 years observed that diets low in fiber increase the risk of CMD, dental caries, and large bowel conditions such as cancer, appendicitis and diverticulosis [65]. Today nutrition guidelines encourage increased consumption of vegetables, fruit, and whole grains [53–56]. Diets rich in whole grains show a strong association with lower risks of most CMD. Risk reduction was greatest when daily intake of dietary fiber was between 25 and 29 g while dose–response curves suggested that higher intakes could confer even greater benefit. Findings from prospective studies and clinical trials associated with relatively high intakes of dietary fiber and whole grains were complementary, and striking dose–response evidence indicates that the relationships to several non-communicable diseases could be causal [64].

### Dietary fats

Dietary fats encompass a highly diverse range of compounds with complex effects on cell physiology, gene expression, and metabolites [66]. They can have vastly different health effects depending on its source (e.g., harmful impact of trans-fats vs beneficial properties of avocado) influenced by accompanying nutrients, molecular lipid structures, and metabolic processing. Reflecting this complexity, merely considering total quantity of dietary fat consumption is not consistently linked to CMD risk across wide ranges (~ 20–40%) of total daily caloric intake [61, 66].

For decades, a low-fat diet was recommended for weight loss and reducing obesity. However, findings from randomized trials have not consistently demonstrated that lowering total fat intake leads to long-term weight loss compared to other dietary interventions [63, 67, 68]. Accordingly, many organizations have concluded that evidence no longer supports an upper limit on total fat consumption [53, 54, 56, 63, 69, 70], except for the World Health Organization, which continues to recommend limiting dietary fat intake to less than 30% [71]. There is, however, consensus among health organizations advocating for the reduction of saturated fatty acids (SFA)—commonly found in animal products and tropical oils (e.g., palm oil, coconut oil, shea butter)—and replacing them with healthy fats, primarily from plant-based foods rich in mono- and polyunsaturated fatty acids (PUFA). Examples include nuts, seeds, and non-hydrogenated vegetable oils. A recent Cochrane review confirmed that reducing saturated fat intake can decrease cardiovascular event risk by 17%. The review also found that replacing SFAs with PUFAs offers greater cardiovascular protection than replacing them with carbohydrates [72]. Another prospective cohort study with up to 45 years of follow-up, involving nearly 80,000 participants, showed that higher SFA intake was strongly associated with increased total and CVD mortality. Conversely, theoretical substitution of SFAs with carbohydrates or monounsaturated fatty acids (MUFA) was linked to a lower risk [73].

In contrast to SFAs, unsaturated fats improve glycemic control, reduce insulin resistance, and lower T2DM risk [49, 66, 74]. While the cardiometabolic benefits of PUFAs are well-established, particularly for omega-3 PUFAs, some scientists argue that omega-6 PUFAs may be harmful [75]. This concern centers on the conversion of linoleic acid to arachidonic acid, a precursor to pro-inflammatory and thrombogenic compounds, which may negatively affect glucose metabolism, weight regulation, and eating behavior [75, 76]. However, a pooled analysis of individual-level data from 20 prospective cohort studies across 10 countries found that linoleic acid provides long-term benefits for T2DM prevention, and arachidonic acid was not associated with harm [76].

Dairy products, including milk, cheese, and yogurt, are notable sources of nutrients and dietary fat. High-fat dairy products have historically been criticized for their saturated fat content and potential CVD risk [77]. However, recent studies suggest a more complex relationship. A systematic review by Giosuè et al. found that moderate dairy consumption does not adversely affect cardiovascular health [77]. While very high milk intake (> 1000 g/day) was linked to increased CVD risk, moderate consumption of fermented milk, butter, and cheese was associated with reduced risk. Cream showed no significant association [78]. Yogurt, in particular, has been consistently linked to lower long-term weight gain [61] and reductions in inflammation [79]. Nonetheless, the evidence surrounding dairy fat and CVD remains mixed, highlighting the need for further research to elucidate underlying mechanisms and achieve consensus.

Similarly, eggs are a topic of debate regarding their cardiovascular impact. Eggs are rich in phospholipids and cholesterol. A study by Dehghan et al. involving 177,000 participants from 50 countries found no significant effect of egg consumption on blood lipid levels or CVD risk [80]. Additionally, a randomized clinical trial by Gálvis et al. demonstrated that consuming two eggs daily did not adversely affect key CVD biomarkers [81]. However, egg yolk phosphatidylcholine has been linked to increased trimethylamine-N-oxide levels, a compound associated with higher CVD risk [81]. More clinical studies with robust patient stratification are needed to clarify the role of eggs in cardiovascular health.

In conclusion, while saturated fats should be limited, emerging evidence suggests that dietary cholesterol, particularly from sources like eggs, may not have the harmful effects previously assumed, especially when consumed in moderation. Current dietary recommendations are shifting from focusing on isolated macronutrients to emphasizing overall dietary patterns, particularly plant-based diets rich in unsaturated fats, which have been associated with improved lipid profiles and reduced blood pressure.

### Dietary proteins

Dietary protein plays a central role in human health, influencing various physiological processes and health outcomes. Proteins are composed of amino acids, which are essential for synthesizing new proteins and fulfilling multiple metabolic functions. Numerous studies highlight the importance of dietary protein in regulating metabolic pathways, including those related to muscle synthesis, inflammation, and satiety. These effects are mediated primarily through signaling pathways involving glucagon-like peptide 1 (GLP-1), peptide YY (PYY), insulin, and leucine-induced activation of mTORC1, which stimulates skeletal muscle protein synthesis following protein-containing meals [82].

The Recommended Dietary Allowance (RDA) for adults is 0.8 g of protein per kilogram of body weight per day [52]. However, recent research suggests that higher protein intake is associated with various health benefits, including improved muscle mass and strength, particularly in older adults. A meta-analysis of 49 studies found that increasing dietary protein intake to up to 1.6 g/kg/day, combined with strength training, significantly improves muscle mass and strength compared to strength training alone in generally healthy, middle-aged, and older populations [83]. Another meta-analysis of 17 randomized controlled trials (RCTs) confirmed these findings, showing that protein supplementation combined with resistance training can mitigate aging-related declines in muscle mass and strength in older individuals [84].

On the other hand, high protein intake has been linked to a modestly elevated risk of T2DM [85, 86]. Animal studies have demonstrated that protein restriction, independent of calorie intake, can extend lifespan in mice and improve the health of young and middle-aged rodents, with potential implications for humans [87]. In non-restrictive feeding strategies, diets with increased protein levels increase growth hormone (GH) signaling and insulin-like growth factor 1 (IGF-1) levels which may shorten rodent lifespans by activating a pro-aging axis [88]. Some human and animal studies suggest that a low-protein diet during middle age may help prevent cancer and improve overall mortality, potentially through the regulation of circulating IGF-1 and insulin levels [89].

The source of dietary protein—whether from animal or plant origins—also appears to have significant health implications, as different protein sources elicit distinct metabolic responses. The amino acid profiles of meat, eggs, and dairy are more closely aligned with human requirements than those of plant foods. Thus, in conditions of food scarcity, it may be easier to achieve protein adequacy through animal-based foods. However, contrary to common misconceptions, all plant foods contain all essential proteinogenic amino acids [90]. For individuals with access to a reasonably diverse diet, daily protein requirements can be easily met [90]. Mixed diets containing 90% of protein from a variety of plant foods and only 10% from animal sources can meet protein needs similarly to typical Western diets, which are significantly higher in animal protein [90, 91]. High intake of animal protein, particularly from processed meat, has been associated with an increased risk of T2DM [92], whereas this risk does not appear to extend to plant proteins [85, 86]. In fact, higher plant protein intake may even improve metabolic health [93]. Current evidence suggests avoiding processed meats and limiting unprocessed red meats and poultry to 1–2 servings per week to optimize metabolic health [61, 92].

### Food processing

Ultra-processed foods (UPFs), such as processed meats, refined grains and sugars, are convincingly linked to metabolic harms [61, 94]. However, most foods require some form of processing to be suitable for human consumption—e.g., chopping, cooking, smoking, freezing, or salting. The NOVA classification system provides a standardized approach to categorizing foods based on their processing level [95]. It divides foods into four main groups: unprocessed or minimally processed foods, processed culinary ingredients, processed foods, and UPFs. UPFs undergo extensive processing and typically contain multiple ingredients, including additives, preservatives, emulsifiers, dyes, color stabilizers, flavorings, and other synthetic substances. They are designed to be hyper-palatable, convenient, and have a long shelf life. UPFs are often ready-to-eat or ready-to-heat products and are characterized by high levels of refined carbohydrates, saturated fats, and salt, while being low in essential nutrients such as fiber [95].

A well-known crossover metabolic ward RCT by Hall et al. examined the effects of UPFs on energy intake and weight [96]. In this study, 20 adults consumed minimally processed and ultra-processed diets ad libitum over two 2-week periods. Meals were designed to be matched for calories, energy density, macronutrients, sugar, sodium, and fiber. Participants consumed 508 kcal/day more on the UPF diet, resulting in a weight gain of 0.9 kg, whereas they lost 0.9 kg when switched to the minimally processed food (MPF) diet. A similar, smaller study from Japan found that overweight or obese individuals randomized to a UPF diet consumed 813 kcal/day more than those on a non-UPF diet, gaining 1.1 kg in just 1 week [97]. Interestingly, in both studies, the increased energy intake was due to greater consumption of carbohydrates and fats, but not protein. This finding supports the protein leverage hypothesis, which suggests that individuals consuming UPFs may overeat in an effort to meet protein requirements [98].

Most evidence linking UPF intake to obesity and CMD comes from observational studies. Multiple meta-analyses indicate that high UPF intake is associated with negative effects on weight management and CMH [99–101], a trend not observed with other NOVA food groups [50, 101]. While the mechanisms by which UPFs contribute to obesity are numerous, they remain inconclusive [101].

### Artificial sweeteners

Artificial sweeteners (AS) have been widely introduced into the food supply over the past few decades as a strategy to reduce sugar and calorie intake. A large meta-analysis found that substituting AS for sugary beverages was associated with small improvements in body weight and CMH [102]. AS are generally considered safe by regulatory agencies [103]; however, their long-term health effects remain unclear, and in 2022, the World Health Organization (WHO) issued guidelines advising against the use of AS [104, 105]. Recent studies have raised concerns about potential health risks associated with AS. One study found that plasma levels of erythritol, a widely used AS from the sugar alcohol family, were a strong predictor of major adverse cardiovascular events within 3 years [106]. Additionally, a large prospective observational study demonstrated a significant association between the consumption of several AS (including aspartame, acesulfame potassium, and sucralose) and an increased risk of cardiovascular events over a median follow-up period of 9 years [107]. Another study by the same group reported a positive association between AS intake and an elevated risk of T2DM [108].

In conclusion, recent evidence suggests that while replacing sugars with AS can lead to short-term reductions in body weight and may lower the risk of dental caries, their long-term use has been associated with an increased risk of T2DM, CVD, and mortality [105].

### Chocolate, coffee and tea

Dark chocolate and cocoa-based products may be beneficial for CMH due to their high flavonoid content, which can exert antioxidant, antihypertensive, anti-inflammatory, and anti-atherogenic effects [109]. A recent meta-analysis showed that calorie-balanced increases in chocolate consumption were associated with lower overall, cardiovascular, and cancer mortality, with the primary benefit mediated by reduced blood pressure [110]. A Mendelian randomization study also found dark chocolate to be beneficial for hypertension and thromboembolism, though no association was observed with ten other CVDs [111]. Observational studies suggest that chocolate consumption may protect against insulin resistance [112] and abdominal obesity [113]. However, recent large meta-analyses, while generally highlighting the benefits of dark chocolate for CMH, note that the credibility of evidence is very low or low, emphasizing significant uncertainty regarding chocolate–disease associations [110, 114–117].

Coffee and tea are widely consumed beverages rich in caffeine and other bioactive compounds [118, 119]. The relationship between coffee consumption and the risk of CHD has been studied for over 60 years [120]. While early case–control studies suggested a positive association between coffee consumption and CHD risk, later prospective studies and meta-analyses have generally found no definitive association [121]. A 2013 cohort study reported that consuming four cups of coffee per day was linked to increased mortality [122]. However, a 2014 meta-analysis of prospective studies concluded that moderate coffee consumption was inversely associated with CVD risk, with the lowest risk observed at 3–5 cups per day [121]. These findings were corroborated by a recent prospective study involving over 170,000 individuals, which found that habitual caffeine intake, particularly at moderate levels, was associated with a lower risk of new-onset CMD [123]. Epidemiologic studies on caffeine intake are often confounded by factors such as smoking and other lifestyle behaviors. Early studies that did not adequately control for these biases led to misleading results, and residual confounding remains a concern even in more recent studies [119].

Given that caffeine is a purine derivative, its effects on serum uric acid levels, hyperuricemia, and gout are of interest. Several studies indicate that coffee consumption is associated with a lower risk of hyperuricemia [124]. For instance, a meta-analysis of observational studies found that higher coffee intake was linked to reduced serum uric acid levels and a lower risk of gout, particularly in men [125]. The effect appears to be dose-dependent, with greater benefits observed at higher levels of coffee consumption. Proposed mechanisms include coffee’s ability to enhance uric acid excretion via the kidneys and the antioxidant effects of chlorogenic acid, a major polyphenol in coffee. Interestingly, studies suggest that these uric acid-lowering effects are likely attributable to non-caffeine components, as similar benefits are observed with decaffeinated coffee [126].

A substantial body of evidence indicates that moderate caffeine intake is consistently associated with a reduced risk of chronic diseases and can be part of a healthy lifestyle.

### Alcohol

Alcohol induces acute anxiolytic effects in humans and has historically been used for this purpose [127]. While excessive alcohol-intake is a well-established risk factor for CMD, the association between mild-to-moderate alcohol consumption and CMH is complex.

Mild-to-moderate alcohol intake increases fatty acid oxidation and glucose uptake by muscle cells and may increase HDL-cholesterol, though the link to improved reverse cholesterol transport is controversial. Conversely, excessive alcohol consumption inhibits oxidation of fatty acids in the liver, leading to fatty acid accumulation, hypertriglyceridemia and eventually impairs glucose uptake increasing insulin resistance [128].

A majority of epidemiological studies suggest that mild-to-moderate alcohol consumption is associated with lower adverse cardiovascular events [127] and lower all-cause and cardiovascular mortality [129], but can increase the risk for cancer [127], atrial fibrillation, hemorrhagic stroke, adiposity and hypertension [53, 130]. Evidence supporting the protective effects of mild-to-moderate alcohol consumption is inconsistent and dietary guidelines discourage alcohol intake for CVD prevention [53, 55].

### Evidence regarding current popular diets

The Dietary Reference Intakes (DRI) include an Acceptable Macronutrient Distribution Range (AMDR) [52] recommendation that outlines a broad range of macronutrients for healthy nutritional intake: carbohydrates, 45–65%; fat, 20–35%; and protein, 10–35%. However, several popular dietary patterns fall outside these ranges, which may cause confusion. For example, very low-fat and ketogenic diets are lower in fat and carbohydrates, respectively, while Mediterranean diet can exceed the fat range, particularly due to its emphasis on extra-virgin olive oil. Compared to usual diet, popular diets generally can slightly improve cardiometabolic parameters at 6 month but differences largely disappear at 12 month, implying that people can choose the diet they prefer [131]. In the following, we aim to clarify the intended implementation of some of the most popular dietary patterns and summarize the evidence regarding their effects on CMH.

### Mediterranean

The Mediterranean diet is one of the most popular and is commonly ranked as one of the healthiest [132–134]. It has been shown to reduce the risk of heart disease, metabolic syndrome, and T2DM [135]. A landmark study followed 7,447 individuals at high cardiovascular risk over 4.8 years and showed a 30% lower incidence of major cardiovascular events among participants following the Mediterranean diet supplemented with extra-virgin olive oil or nuts, as compared to those assigned to a reduced-fat diet [132]. The group supplemented with extra-virgin olive oil aimed to consume ≥ 50 g/day of the polyphenol-rich olive oil, while those supplemented with nuts, were recommended to consume 30 g of nuts, composed of 15 g of walnuts, 7.5 g of almonds, and 7.5 g of hazelnuts. Recommendations for both mediterranean diets included optional Wine with meals for habitual drinkers (≥ 7 glasses/week).

Meta-analysis comparing different popular diets found Mediterranean diet as most effective dietary approach to improve glycemic control in patients with T2DM [136] and LDL cholesterol in general [131]. A systematic review ranked the Mediterranean diet as the most likely dietary model to provide protection against CHD [137]. The traditional Mediterranean diet is characterized by a high intake of olive oil, fruits, nuts, vegetables, and cereals; a moderate intake of fish and poultry; and a low intake of dairy products, red meat, processed meats, and sweets. It also can include wine in moderation, consumed with meals [132]. Guidance on sodium varies across different sources and includes both implicit and explicit recommendations (e.g., many Mediterranean patterns implicitly limit sodium by restricting the consumption of processed foods, which are a primary contributor of sodium). Recommendations for dairy foods vary across sources, with some advising limits and others advocating for the inclusion of yogurt and cheese, with little emphasis on differentiating between low-fat and whole-fat options. This dietary pattern is unique in its inclusion of moderate alcohol consumption [49].

The health effects of individual foods, together with the above results, provide strong evidence supporting a Mediterranean-type diet for CMH.

### DASH (dietary approaches to stop hypertension)

This dietary pattern is based on a large multicenter trial published in 1997, which examined the impact of dietary patterns on blood pressure in 459 adults. The study showed that a diet rich in fruits, vegetables, and low-fat dairy, with reduced saturated and total fats, can substantially lower blood pressure [138]. The sodium chloride content of each diet was similar—approximately 3 g per day. Relative to the control diet, it reduced systolic blood pressure by 5.5 mmHg and diastolic blood pressure by 3.0 mmHg. In 133 participants with hypertension, the diet produced a net reduction in systolic and diastolic blood pressure of 11.4 and 5.5 mmHg, respectively—a reduction similar in magnitude to that observed in trials of drug monotherapy for hypertension [138]. It was estimated that a population-wide reduction in blood pressure of this magnitude would reduce the incidence of coronary heart disease by ~ 15% and stroke by ~ 27% [138]. In a recent scientific statement from the American Heart Association (AHA) comparing popular diets, DASH-style patterns aligned best with AHA criteria, followed by the Mediterranean diet [49].

### Plant-based and portfolio diet

Vegetarian-style dietary patterns generally restrict animal products. There are several types of vegetarian diets, including Pescatarian (which excludes meat and poultry but includes fish, dairy, and eggs), Lacto-ovo-vegetarian (excludes meat, poultry, and fish), and vegan (excludes meat, poultry, fish, dairy, and eggs) [49].

A recent study that followed over 400,000 Americans for 20 years found that a diet with primarily fat from plant sources was associated with decreased mortality. Animal fat, on the other hand, was associated with an increased risk of death in this study [6]. However, distinguishing between animal and plant fats may be an oversimplification, as it is important to consider the composition. For example, fish fat, which provides important omega-3 fatty acids, is considered animal fat, while palm and coconut fats, although plant-based, contain unhealthy long-chain saturated fats.

Another large prospective cohort study, including over 70,000 Seventh-day Adventists between 2002 and 2007, found vegetarian diets to be associated with lower all-cause mortality compared to non-vegetarian diets [139]. It also found some associations with lower mortality for pescatarian, vegan, and lacto-ovo–vegetarian diets specifically, compared to non-vegetarian diets [139]. Vegetarian dietary patterns have also been associated with a lower risk of metabolic syndrome [140, 141] and a protective effect against T2DM, while pesco- and semi-vegetarian diets offered intermediate protection [141].

A specific form of plant-based diet is the Portfolio Diet, designed by David Jenkins to lower cholesterol and improve cardiovascular and metabolic health by combining known cholesterol-lowering foods. Initially tested on himself, it later showed in a small RCT of 46 healthy hyperlipidemic adults a maximal LDL-C lowering efficacy similar to that of 20 mg lovastatin (− 28.6% versus − 30.9%) in a “head-to-head” comparison when all foods were provided under metabolically controlled conditions [29]. Key components include plant sterols (phytosterols, found in fortified foods), viscous fiber (from oats, barley, and legumes), soy protein (from tofu and soy milk), and nuts. A subsequent longer-term multi-center RCT, including 351 participants with hyperlipidemia, showed smaller reductions of 10–15% under free-living conditions, with adherence only at 43% of the initial metabolic trial, as participants received only dietary advice [142]. In a systematic review and meta-analysis of seven trial comparisons, the Portfolio Diet significantly reduced LDL-C by ~ 17%, as well as apolipoprotein B, total cholesterol, triacylglycerols, blood pressure, and C-reactive protein. There was no effect on HDL-C or body weight [143].

It is essential to recognize that the quality of plant-based foods consumed plays a critical role in determining health outcomes [144]. One prospective investigation of a plant-based diet and the incidence of CVD found that healthier plant foods (i.e., whole grains, fruits, vegetables, nuts, legumes, oils, tea, and coffee) were associated with improved metabolic health, whereas less-healthy plant foods (i.e., fruit juices, sweetened beverages, refined grains, potatoes/fries, and sweets) were associated with an increased incidence of CVD [145].

In summary, a healthy plant-based diet emphasizes plant foods and minimizes animal products, offering numerous health benefits while promoting environmental sustainability.

### Low-carbohydrate and ketogenic diets

Generally, low-carbohydrate (low-carb) diets produce similar or greater weight loss than low-fat diets, with corresponding improvements in blood pressure and lipid profiles [62]. A large 12-month weight loss study comparing healthy low-carb versus healthy low-fat diets showed no significant difference in weight loss. However, both groups experienced significant weight loss (− 5.3 kg for the low-fat diet versus − 6.0 kg for the low-carb diet) [68]. The focus of healthy low-carb diets is to reduce exposure to carbohydrates, especially ultra-processed foods rich in refined starches and sugars. This approach may explain why low-carb diets are the most effective for HbA1c reductions in a meta-analysis of dietary approaches [136]. Other meta-analyses further suggest that low-carb diets may be superior to low-fat diets for glycemic control in patients with T2DM [146, 147]. These diets emphasize vegetables, fruits, nuts and seeds, fish and seafood, and non-tropical oils, while limiting carbohydrate intake to 30–40% of daily calories. They focus on whole grains, legumes, and dairy, while minimizing foods rich in refined starches and sugars [49].

An extreme form of low-carb diet is the ketogenic diet, which consists of a high-fat component, very low carbohydrates, and adequate protein intake. Typically, energy intake from dietary carbohydrates is limited to less than 10% of daily intake [148, 149]. The ketogenic diet has been clinically used since the early 1920s to control seizures in patients with epilepsy [150]. Recently, interest in the ketogenic diet has increased due to its beneficial effects in a number of diseases, including obesity [61, 151, 152], T2DM [151], high blood pressure [153], and cancer [149].

One study compared 262 diabetic patients undergoing a ketogenic diet to usual care over 1 year. The ketogenic diet led to a reduction in HbA1c from 7.6 to 6.3%, a 12% loss in body weight, and a significant reduction in medication use. These improvements occurred safely while dyslipidemia and markers of inflammation and liver function also improved [152]. However, a 2019 review of available data found that most improvements relative to a comparison diet were no longer significant after 12 months [154]. An elegant metabolic-ward study showed that participants on a cross-over ad libitum ketogenic animal-based diet consumed nearly 700 kcal more per day compared to a plant-based version, but there was no difference in weight gain [155]. Some challenges of the ketogenic diet include initial symptoms such as headache, fatigue, irritability, nausea, and constipation, which improve over time. However, the lack of benefits from fruits, vegetables, beans, legumes, and whole grains [49, 61] has led to official discouragement of the routine use of the ketogenic diet [54]. It may be most useful for initial weight loss (e.g., over 6–12 months), after which minimally processed, bioactive-rich foods can be gradually reintroduced. Potential long-term health effects require further investigation [61].

### Paleo diet

The Paleo diet, or Paleolithic nutrition [156], is based on the idea that the typical food of late Paleolithic humans consisted of lean wild meats, fruits and non-starchy vegetables, which was very different from the modern diet that includes dairy, large portions of cereals, and highly processed foods [156]. The human diet evolved much more rapidly than our genetics, which developed before the advent of agriculture. Therefore, Paleolithic foods may be better suited to our pre-agricultural energy metabolism than the current modern diet [157]. A systematic review of four randomized controlled trials, involving 159 participants with one or more components of metabolic syndrome, found that Paleo diet resulted in greater short-term improvements in metabolic syndrome components compared to guideline-based control diets [158]. However, scientific evidence supporting the long-term health benefits of the Paleo diet is still lacking. A more recent systematic review and meta-analysis, which included 98 participants, found no evidence of greater health benefits from the Paleolithic diet compared to other healthy diets regarding glucose and insulin homeostasis in individuals with altered glucose metabolism [159].

## Emerging areas

### Intermittent fasting and time-restricted eating

Caloric restriction (CR) has been shown to extend lifespan in multiple species [160, 161] and has well-established benefits in CMH for humans, but many individuals find it difficult to sustain CR over the long-term. Intermittent fasting (IF), an alternative form of dietary restriction that may or may not include CR, can also improve CMH and is potentially more sustainable in humans. Interest in IF has been growing, with both animal and human studies exploring its effects. There are different forms of IF, ranging from time-restricted eating (TRE) with a limited number of hours, to alternate-day fasting, or fasting for 2 days per week. The most common form of IF typically involves 12–23 h of fasting per day [51, 88].

The reported beneficial effects of IF, particularly through a pattern of TRE, are based on improvements in metabolic balance by stabilizing circadian rhythms and initiating a process called metabolic switching [162]. A metabolic switch occurs when a negative energy balance is reached, usually at least 12 h after stopping food intake. At this point, hepatic glycogen stores are depleted, and fatty acids are released from adipose tissue through lipolysis. The metabolic shift from glucose metabolism to the use of fatty acids and ketones represents an evolutionarily conserved mechanism. This shift redirects metabolism from lipid and cholesterol synthesis and storage to the mobilization of fat through fatty acid oxidation and ketone production, helping preserve muscle mass and function [162, 163].

At the cellular level, the delayed aging phenotype associated with IF is linked to increased metabolite recycling, autophagy, and enhanced maintenance and repair mechanisms tied to stress response pathways. The overall result is a reprogrammed metabolism with improved repair and recycling processes, with improved inflammation and oxidative stress [164, 165] as well as reduced growth and macromolecular synthesis [87, 88, 162].

Nonetheless, the extent to which these mechanistic pathways confer clinical advantages independent of weight loss remains uncertain.

Most human clinical trials on IF have focused on weight loss or improvements in cardiometabolic biomarkers, typically involving subjects with CMD and using an 6–12 h daily eating window [166–172].

A landmark 12-month human RCT involving 139 participants with obesity compared TRE with CR or daily CR alone. It found similar improvements in weight loss (− 8.0 kg for the TRE group vs − 6.3 kg for the CR group) and metabolic parameters, suggesting that CR accounted for most of the improvements seen with TRE [173]. Similarly, another major 12-week human trial with 116 adults (BMI > 25) found that TRE, in the absence of other interventions, was not more effective for weight loss than eating throughout the day [174]. A more recent large RCT over 12 weeks including 197 adults with overweight or obesity compared early-, late- and self-selected 8 h TRE to usual care (education about the Mediterranean diet). Visceral adiposity measured by magnetic resonance imaging (primary outcome) was similar in all four groups. However, all TRE groups decreased the energy intake by 300–500 kcal daily, which led to an average weight loss of approximately 3 kg compared with the usual care group. Furthermore, they observed a significant decrease in fasting glucose levels in the early TRE group compared with the other three groups [175].

Other smaller studies found that IF can lead to modest weight loss without significant CR [176–178]. One small study even reported a reduction in caloric intake by 550 kcal/day without calorie counting [179].

A meta-analyses of 13 randomized controlled/comparison trials with matched energy intakes (isocaloric) between IF and CR found similar benefit in CMH compared with CR, with very limited evidence suggesting that IF may be more effective versus CR for fat loss and insulin sensitivity [167]. Another meta-analysis of 7 studies found that addition of TRE to CR produced greater weight loss and improvements in CMH in 3 of the 7 studies [166]. When no CR is intended however, TRE appeared to be more effective for weight loss and improved CMH compared to other diet interventions in a meta-analyses of 12 studies; moreover early TRE was more beneficial for weight loss and CMH than delayed TRE [171].

Another recent meta-analysis found that TRE significantly improved CMH parameters, identifying an optimal feeding window of 6–12 h, differentiated for specific parameters, with the last eating time point at 6–8 PM [172]. Another group confirmed these findings in their meta-analysis, reporting that a TRE regimen of 6–10 h over 5–14 weeks improved glycemic parameters in individuals with overweight, obesity, or T2DM. Eating early in the day provided greater benefits for some glycemic parameters [170].

In conclusion, TRE appears to be a safe and effective dietary approach to improve CMH and weight reduction in adults with CMD compared with ad libitum eating. Early TRE may be more beneficial for glucose homeostasis than delayed TRE. When directly compared with CR the results are less clear, yet the addition of TRE to CR interventions can produce greater weight loss and improvements in CMH in some circumstances. Additional research is needed to further clarify the specific circumstances in which the addition of TRE does or does not provide benefits to CR interventions.

### Gut microbiome

Traditionally, “germs” have been associated with infection and disease, but in the past decade, there has been an explosion of interest and research on the microbiota and its important role in protecting against pathogens through the “education” of the immune system [180], as well as its involvement in multiple processes related to cardiometabolic and neurological health [180].

Fecal transplantation from obese female twins into germ-free mice led to increased total body and fat mass, as well as obesity-associated metabolic phenotypes. In contrast, mice receiving microbiota from the lean co-twin prevented the development of these changes [181]. Another experiment found that infusion of gut microbiota from lean donors improved insulin resistance in patients with metabolic syndrome [182]. These early experiments highlighting the importance of gut microbiome have recently been translated into human, double-blind RCTs. Recently, microbiota-directed food interventions have been found to be an effective dietary supplementation strategy for undernourished children [183–185]. Two RCTs showed significant improvements in glucose metabolism in adults [186, 187].

### Practical recommendations for cardiometabolic health

Despite the often striking differences in recommendations across dietary patterns, high-quality diets from all approaches tend to converge on similar principles. There is near-universal agreement to prioritize vegetables and whole foods, while reducing or avoiding sugar and refined grains. There is also widespread consensus that fruits, nuts, seeds, fish, and healthy vegetable oils can be integral components of a healthy diet and are consistent with current nutrition guidelines [53].

Focusing on improving adherence to these shared recommendations would be a significant step forward in reducing the prevalence of CMD.

## Data Availability

No datasets were generated or analysed during the current study.

## Abbreviations

AHA: American Heart Association
AMDR: Acceptable macronutrient distribution range
AS: Artificial sweeteners
CETP: Cholesteryl ester transfer protein
CHD: Coronary heart disease
CMD: Cardiometabolic disease
CMH: Cardiometabolic health
CVD: Cardiovascular diseases
DASH: Dietary approaches to stop hypertension
DRI: Dietary reference intakes
GH: Growth hormone
GLP-1: Glucagon-like peptide 1
HCAR: Hydroxycarboxylic acid receptors
HDL: High-density lipoprotein
IF: Intermittent fasting
IGF-1: Insulin-like growth factor 1
LDL: Low-density lipoprotein
MPF: Minimally processed food
MUFA: Monounsaturated fatty acid
PUFA: Polyunsaturated fatty acid
PYY: Peptide YY
RCT: Randomized controlled trial
RDA: Recommended dietary allowance
SFA: Saturated fatty acids
T2DM: Type 2 diabetes mellitus
TRE: Time-restricted eating
UPF: Ultra-processed food
WHO: World Health Organization
